# Comprehensive analysis of the efficacy and safety of CAR T-cell therapy in patients with relapsed or refractory B-cell acute lymphoblastic leukaemia: a systematic review and meta-analysis

**DOI:** 10.1080/07853890.2024.2349796

**Published:** 2024-05-13

**Authors:** Sebastian Emmanuel Willyanto, Yohanes Audric Alimsjah, Krisanto Tanjaya, Aekkachai Tuekprakhon, Aulia Rahmi Pawestri

**Affiliations:** a Bachelor Study Program of Medicine, Faculty of Medicine, Universitas Brawijaya, Malang, Indonesia; b Institute of Immunology and Immunotherapy, University of Birmingham, Birmingham, United Kingdom; c Department of Parasitology, Faculty of Medicine, Universitas Brawijaya, Malang, Indonesia

**Keywords:** CAR T-cell therapy, immunotherapy, acute lymphoblastic leukaemia, targeted therapy, oncoimmunology

## Abstract

**Background:**

Relapse/refractory B-cell acute lymphoblastic leukaemia (r/r B-ALL) represents paediatric cancer with a challenging prognosis. CAR T-cell treatment, considered an advanced treatment, remains controversial due to high relapse rates and adverse events. This study assessed the efficacy and safety of CAR T-cell therapy for r/r B-ALL.

**Methods:**

The literature search was performed on four databases. Efficacy parameters included minimal residual disease negative complete remission (MRD-CR) and relapse rate (RR). Safety parameters constituted cytokine release syndrome (CRS) and immune effector cell-associated neurotoxicity syndrome (ICANS).

**Results:**

Anti-CD22 showed superior efficacy with the highest MRD-CR event rate and lowest RR, compared to anti-CD19. Combining CAR T-cell therapy with haploidentical stem cell transplantation improved RR. Safety-wise, bispecific anti-CD19/22 had the lowest CRS rate, and anti-CD22 showed the fewest ICANS. Analysis of the costimulatory receptors showed that adding CD28ζ to anti-CD19 CAR T-cell demonstrated superior efficacy in reducing relapses with favorable safety profiles.

**Conclusion:**

Choosing a more efficacious and safer CAR T-cell treatment is crucial for improving overall survival in acute leukaemia. Beyond the promising anti-CD22 CAR T-cell, exploring costimulatory domains and new CD targets could enhance treatment effectiveness for r/r B-ALL.

## Introduction

Acute lymphoblastic leukaemia (ALL) is a malignancy occurring predominantly in childhood, with the peak age of diagnosis between 2 and 10 years of age. It is responsible for 72% of all cases of paediatric leukaemia and one-fourth of all paediatric malignancies [1]. Based on the Global Burden of Disease in 2019, it is reported that the incidence rate of ALL in overall population was 1.96 per 100,000 people per year with a mortality rate of 0.63 per 100,000 people per year [2].

With novel developments in chemotherapy, haematopoietic stem cell transplant, and supportive care, the long-term survival for paediatric ALL has increased to 85–90%. Nevertheless, the relapse rate (RR) in developed countries was still 15–20% in the past two decades. This high RR remains an issue due to the lack of standard treatment for patients with relapse/refractory B-cell ALL (r/r B-ALL), which also reduces the long-term survival to 30–60% [3]. Therefore, the development of efficacious modalities to improve the long-term survival of patients with r/r B-ALL is critical.

Recently, therapeutic advancements have been made, particularly in immunotherapy. Contrary to conventional adaptive immune cells, immunotherapy employs modified cells in a specific targeted therapy to eliminate cells expressing relevant tumour-associated antigens. The advent of chimeric antigen receptor (CAR) T-cell therapy has demonstrated promising results in r/r B-ALL. The principal advantage of CAR T-cell therapy is the high specificity, leading to reduced occurrence of side effects which are beneficial for the recovery process of the patients post-treatment compared to conventional treatments. CAR T-cells use the autologous native T-cells that have been genetically modified *ex vivo* to selectively detect extracellular tumour antigens. Utilizing the advantages of adaptive immunity, CAR T-cell therapy combines the antigen specificity of antibodies with the cytotoxic function of T-cells. T-cell activation requires the engagement of the endogenous T-cell receptor (TCR) by its cognate peptide/major histocompatibility complex (MHC) [4]. Activated T-cells will then be directed against lymphoblastic B-cells expressing several targets [5]. CAR T-cell is classified into five different generation based on their intracellular signalling domain, which may include CD3 zeta (CD3ζ) [1st generation], plus a costimulatory domain (4-1BB or CD28) to enhance T-cell activation [2nd generation] or multiple costimulatory domains (4-1BB or CD28, CD134 or CD137) [3rd generation]. Furthermore, the mechanism of tumour termination also led to further CAR T-cell generation, such as IL-12 secretion and the incorporation of the inducible caspase 9 (iCasp9) suicide gene in the 4th generation CAR T-cell therapy to overcome tumour immunosuppression, and the 5th generation CAR T-cell therapy which is currently under development [4].

The main target of CAR T-cell therapy for B-ALL is CD19, a highly expressed surface biomarker of the B-cell lineage. It is responsible for B-cell activation, thus controlling the growth of malignant lymphoblastic cells in ALL. CAR T-cells directed towards CD19 result in the targeted destruction of lymphoblastic cells. CD22, which is also required for the development and differentiation of lymphoblasts, has been offered as an alternative CAR T-cell target for treating r/r B-ALL for patients who are unsuited for CD19 CAR T-cell therapy or relapse cases due to the loss/downregulated expression of CD19 on the cell surface [6]. Furthermore, the incorporation of a costimulatory domain target into CAR T-cell therapy has several advantages. The costimulatory signalling is specifically delivered to T-cells that express the CAR and are exposed to the target antigen, thus enhancing the precision of therapy. Furthermore, the costimulatory signals are not reliant on the tumour or its microenvironment to express costimulatory receptor ligands, ensuring that the therapy can work independently of the surrounding conditions [7].

Currently, there are six FDA-approved CAR T-cell therapies [8]. Among them, KYMRIAH^®^ (tisagenlecleucel) and TECARTUS™ (brexucabtagene autoleucel) were approved for r/r B-ALL [9], the latter of which was FDA-approved for adult B-ALL [10]. KYMRIAH^®^, a second-generation CAR T-cell product (4-1BB costimulatory domain) directed against CD19, is indicated for both paediatric and adult ALL patients and will be one of the focuses of this study. It is the first CAR T-cell therapy to receive FDA approval with an overall remission rate of 81% in children and young adults with r/r B-ALL [8]. However, the treatment is still controversial due to relapses in some individuals and the occurrence of fatal adverse events, including the cytokine release syndrome (CRS) and immune effector cell-associated neurotoxicity syndrome (ICANS). These risk-to-benefit imbalances resulted in the restitution to using haematopoietic stem cell transplant (HSCT) for the treatment of r/r B-ALL [4].

To tackle these controversial treatment outcomes and anticipate the rise of new CAR T-cell modalities, this study aimed to determine the efficacy and safety of CD19, CD22, and the combination of CD19/22 CAR T-cell therapy in paediatric r/r B-ALL management using a meta-analysis approach. We also performed an in-depth analysis regarding the efficacy and safety of various costimulatory domains of CD19 CAR T-cell therapy. Information on the treatment outcomes will provide insights for considerations and selection of employing CAR T-cell therapy. It will also serve as the foundation for further development of CAR T-cell therapy to effectively improve the overall survival of r/r B-ALL patients.

## Methods

### Search strategy

**Figure 1.**
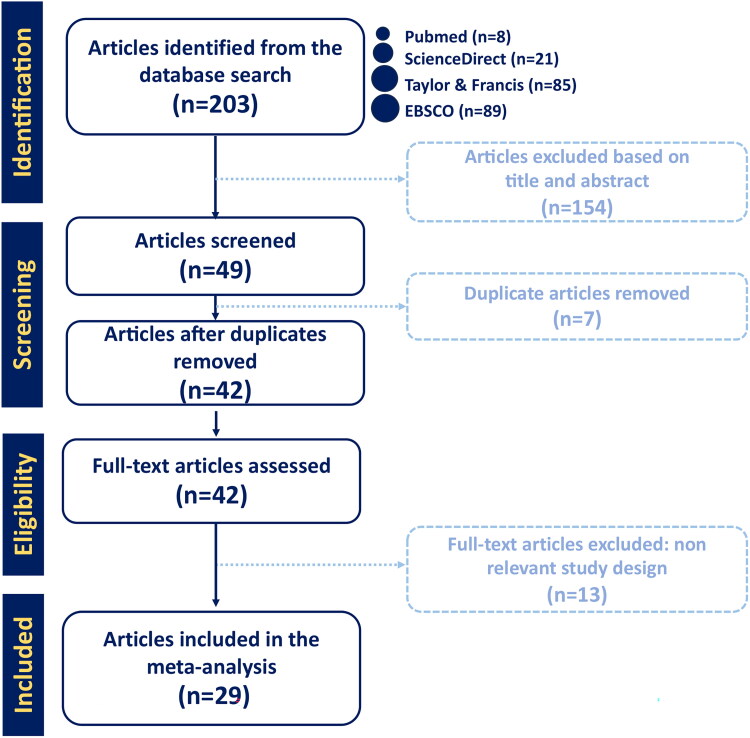
Preferred reporting items for systematic reviews and meta-analyses (PRISMA) flowchart for study identification and selection. The original database search resulted in 203 studies from four databases, including PubMed, ScienceDirect, EBSCO, and Taylor & Francis. Through title and abstract screening, 154 articles were removed and 49 articles were screened for duplication. Duplicate screening resulted in seven removed articles. Forty-two articles were furthermore assessed for eligibility and 12 articles were removed due to irrelevant study design. This step resulted in 29 final studies included in the qualitative synthesis.

This meta-analysis was constructed according to the preferred reporting items for systematic reviews and meta-analyses (PRISMA) statement guidelines [11]. The literature search was carried out with keywords using Boolean operators, including (‘CAR T-cell’) AND (‘Paediatric’) AND (‘ALL’ OR ‘Acute Lymphoblastic Lymphoma’). The literature search was conducted independently by three authors using keywords referring to the PICO framework in Supplementary Table 1. The acquired articles underwent title and abstract screening, followed by duplicate removal, before being assessed thoroughly for eligibility (Figure 1).

To obtain specific and homogenous search results, several inclusion criteria were determined, including (1) peer-reviewed clinical studies, (2) published up to March 2023, (3) available or accessible in English, (4) involving children and/or adolescents (0–18 years old) with refractory B-ALL, and (5) employing CAR T-cell therapy as the intervention of interest. Articles that could not be accessed online and those with preclinical or *in vivo* studies were excluded (Figure 1).

### Determination of outcomes of interest

In this study, we assessed the efficacy and safety of CAR T-cell therapy for r/r B-ALL. The efficacy parameters include minimum residual disease negative complete remission (MRD-CR) and RR. MRD-CR is characterized by the absence of leukaemia cells in the bone marrow by microscopic or sensitive tests, such as flow cytometry or PCR [12]. Finally, RR is described as the rate of recurrence of the disease at any site after a period of complete remission, either while still on or after completion of front-line therapy.

CRS and ICANS grades 3 and 4 were used to determine the safety of CAR T-cell therapy. CRS was described as fatal adverse events appearing 14 days after CAR T-cell infusion due to systemic inflammatory response caused by rapidly activated CAR T-cells [10]. CRS was classified into four grades, of which the 3rd and 4th grades were considered as life-threatening adverse events. ICANS is a severe form of neurotoxicity syndrome often starting at 4–5 days following CAR T-cell infusion. ICANS is further classified into four grades based on the immune effector cell-associated encephalopathy (ICE) score [12].

### Data extraction and risk of bias assessment

Literature search and data extraction were carried out independently by three authors on several databases, including PubMed, ScienceDirect, EBSCO, and Taylor & Francis (Figure 1). The selected studies were extracted and assessed for their eligibility and accuracy by all authors to resolve disagreements on the inclusion. Risk of bias assessment was carried out using the Newcastle–Ottawa Scale (NOS) for non-randomized clinical trial studies (Supplementary Table 2). Three domains of the study quality assessed in NOS included the selection, comparability, and outcome domains [13]. The scoring of each domain was determined by the number of stars assigned to the respective domains following the NOS checklist. The total number of stars described the overall quality of a study, with scores of 6–9, 3–5 and 0–2 considered as low, intermediate, and high-risk biases, respectively.

### Quantitative data analysis

Meta-analysis was conducted using comprehensive meta-analysis (CMA) V3 and Review Manager 5.4 [14, 15]. The meta-analyses results of the included single group studies obtained from CMA were visualized in a forest plot using STATA 17 [16]. The data were evaluated based on the event rates and odds ratios, which were classified as dichotomous data type with 95% confidence interval (CI). Inverse variance model served as the statistical method, while both random and fixed effect models were used during analysis based on the heterogeneity of each outcome. The cut-off of heterogeneity was *I*^2^ > 50% which indicated the use of random effect model for statistical analysis.

## Results

### Study selection, identification and risk of bias assessment

As shown in the PRISMA flowchart (Figure 1), through the literature search, 203 studies published in the last ten years were obtained from four databases (PubMed, ScienceDirect, EBSCO, and Taylor & Francis). After the title and abstract screening, 154 studies were excluded due to unrelatable content. Seven articles were further excluded due to duplication. Forty-two articles passed the initial screening. However, we had to further exclude 13 articles due to irrelevant outcome data (*N* = 7) and unavailable online data (*N* = 6). Finally, 29 studies were included in the qualitative synthesis, while 28 of them were deemed eligible for quantitative analysis [17–43]. One study was not included in the quantitative analysis due to insufficient data.

Out of 29 studies assessed, 26 studies (89.7%) were classified as having low risk of bias [17, 21, 24, 26, 30–41, 43–45], while three studies (10.3%) possessed intermediate risk of bias [19, 21–25, 29, 42]. No high-risk bias studies were found among the included studies. The main bias source arose from the lack of exclusion and inclusion criteria in the methodology and the presence of missing data in the result section (Supplementary Table 2).

### Profiles of included studies

Out of the 29 studies, anti-CD19 CAR T-cell therapy was used in 25 studies (86.20%), anti-CD22 in two studies (6.89%) [37, 38], and the combination of anti-CD19 and anti-CD22 (CD19/22) in two studies (6.89%) [34, 35, 38]. All studies included in the analysis were clinical trials, and among them, 18 studies (62.9%) were treatment trials, seven studies (24.14%) were cohort studies, and three studies (10.3%) were multi-arm multi-stage (MAMS) trials. Ten studies (34.5%) were multi-centred, 17 (58.6%) were conducted only at one centre, while the remaining two (6.9%) had not reported their study sites. The included articles were deemed sufficient to be extracted in this study due to the availability of the outcome of interest data and acceptable risk of bias. Although some of the included studies did not report all outcome parameters, the available data were sufficient for extraction. The detailed summary of the included studies, including the age of subjects, therapeutic intervention and dose, and the region where the study was conducted is available in Supplementary Table 3.

### Patient and intervention characteristics

We included 29 studies enrolling a total sample size of 1367 participants, with a median of 37 (range 4–255) participants. The median age at enrolment was 14.2 (0–30.4) years. Male participants comprised 54.2% (*N* = 553), resulting in a male to female ratio of 1.18. The median duration of study was 23.5 (ranging from 1 to 60) months (Supplementary Table 3).

Most interventions involved CAR T-cells targeting CD19 (25/29, 86.2%), two reported anti-CD22 (6.9%), and two utilized anti-CD19/22 (6.9%). Six studies reported haploidentical haematopoietic stem cell (haplo-HSCT) transplantation pre- or post-CAR T-cell treatment. The commonly used CAR T-cell dose was 1 × 10^6^ cells/kg, although three studies reported doses as low as 0.2 × 10^6^ cells/kg, while one described up to 10 × 10^6^ cells/kg.

### Efficacy of CAR T-cell therapy for r/r B-ALL

#### Minimal residual disease negative complete remission (MRD-CR)-based CAR T-cell efficacy

To assess the efficacy of CAR T-cell therapy, we analyzed outcomes of MRD-CR, characterized by the absence of leukaemia cells in the bone marrow, and the RR. We evaluated the effect size, significance and heterogeneity among groups.

**Figure 2.**
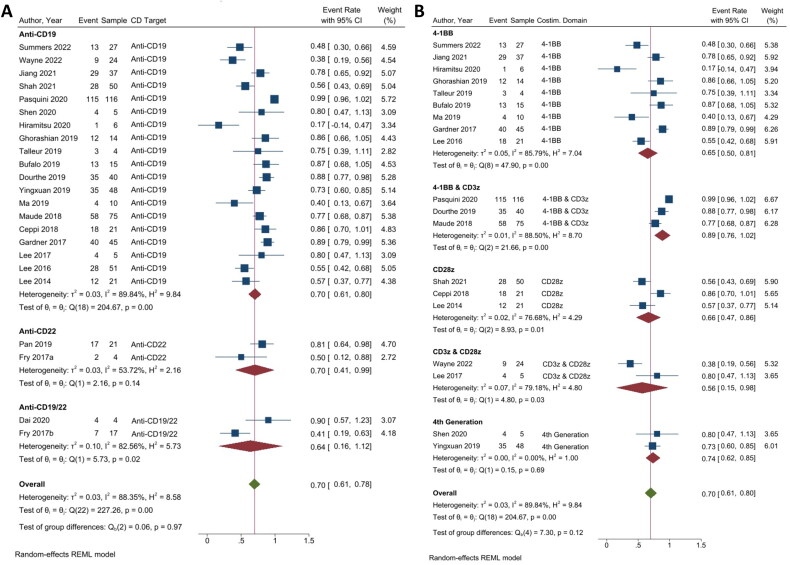
The minimum residual disease negative complete remission (MRD-CR) of CAR T-cell therapy. (A) Subgroup analysis of CD targets for MRD-CR. The analysis included data from 23 studies classified into three CD target subgroups, namely anti-CD19, anti-CD22 and anti-CD19/22. (B) Subgroup analysis of costimulatory domain target groups for MRD-CR. The analysis included data from 19 studies classified into five costimulatory subgroups, namely 4-1BB, 4-1BB & CD3ζ, CD28ζ, CD3ζ & CD28ζ and 4th generation. The *y*-axis indicates the median of the overall pooled estimate. The blue square and solid lines represent event rates with 95% confidence intervals. The size of the squares indicates the weight of each study. The red rhombus indicates the pooled estimate with 95% confidence intervals for each subgroup; meanwhile, the green rhombus indicates the overall pooled estimate.

The analysis of MRD-CR was divided into two categories: those focusing on the most effective CD targets (Figure 2(A)) and those centering on the most effective costimulatory domain (Figure 2(B)). In the context of MRD-CR analysis, a higher event rate will be regarded as a more favourable outcome. To evaluate the MRD-CR of CD targets, 23 studies were analyzed. Within the effective CD targets, three subgroups, namely anti-CD19 (19/23 studies), anti-CD22 (2/23 studies), and the combination of anti-CD19 and anti-CD22 (2/23 studies), were evaluated. Among these subgroups, the anti-CD19 and anti-CD22 groups displayed an equivalent event rate of MRD-CR (ER = 0.70; 95% CI = 0.61, 0.80; *p* < 0.01; *I*^2^ = 89.84% and ER = 0.70; 95% CI = 0.41, 0.99; *p* = 0.14; *I*^2^ = 53.72%) [19, 20], followed by the combination of anti-CD19/22 (ER = 0.64; 95% CI = 0.16, 1.12; *p* = 0.66; *I*^2^ = 82.56%) (Figure 2(A)). The quantitative analysis focusing on anti-CD groups held a significant overall event rate of MRD-CR and a high heterogeneity among the studies (ER = 0.70; 95% CI = 0.61, 0.78; *p* < 0.01; *I*^2^ = 88.35%).

Nineteen studies from the anti-CD19 subgroup were further assessed for the MRD-CR of the costimulatory domain of CAR T-cell therapy. They were subdivided into five subgroups, including 4-1BB (CD137) (10/19 studies), 4-1BB & CD3ζ (4/19 studies), CD 28ζ (4/19 studies), CD3ζ & CD28ζ (3/19 studies), and 4th generation (3/19 studies). The 4-1BB had the highest number of studies due to it being the main costimulatory signal of interest in anti-CD19 CAR T-cell therapy. Out of these five groups, the 4-1BB & CD3ζ had the highest event rate of MRD-CR (ER = 0.89; 95% CI = 0.76, 1.02; *p* < 0.01; *I*^2^ = 88.50%), followed by 4th generation (ER = 0.74; 95% CI = 0.62, 0.85; *p* < 0.01; *I*^2^ = 0.00%), and CD28ζ (ER = 0.66; 95% CI = 0.47, 0.86; *p* = 0.12; *I*^2^ = 76.68%). Lower MRD-CR event rates were found in the 4-1BB and CD3ζ & CD28ζ groups (ER = 0.65; 95% CI = 0.50, 0.81; *p* = 0.04; *I*^2^ = 85.79% and ER = 0.56; 95% CI = 0.15, 0.98; *p* = 0.89; *I*^2^ = 79.18%). All 19 studies from the anti-CD19 subgroup showed a significant overall event rate of MRD-CR and high heterogeneity (ER = 0.70; 95% CI = 0.61, 0.80; *p* ≤ 0.01; *I*^2^ = 89.84%).

#### RR-based CAR T-cell efficacy

**Figure 3.**
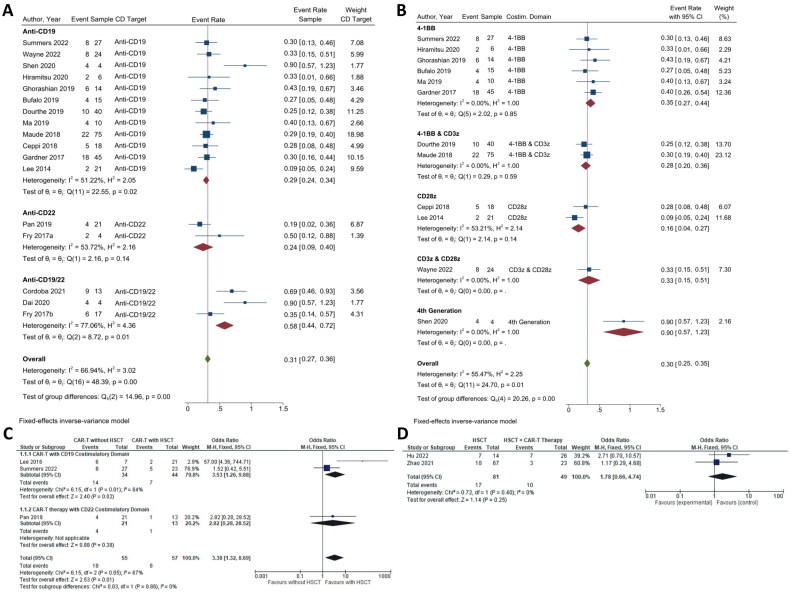
The relapse rate of CAR T-cell CD targets and the CD19 costimulatory domains. (A) Subgroup analysis of CD targets for relapse rate. The analysis included data from 17 studies classified into three CD target subgroups, namely anti-CD19, anti-CD22 and anti-CD19/22. (B) Subgroup analysis of costimulatory domain target groups for relapse rate. The analysis included data from 12 studies classified into five costimulatory subgroups, namely 4-1BB, 4-1BB & CD3ζ, CD28ζ, CD3ζ & CD28ζ, and 4th generation. The *y*-axis indicates the median of the overall pooled estimate. The blue square and solid lines represent event rates with 95% confidence intervals. The size of the squares indicates the weight of each study. The red rhombus indicates the pooled estimate with 95% confidence intervals for each subgroup; meanwhile, the green rhombus indicates the overall pooled estimate. (C) Subgroup analysis of the relapse rate of haplo-HSCT and CAR T-cell therapy. This analysis incorporated information from three studies focused on CAR T-cell therapy, with or without haplo-HSCT, and two studies examining haplo-HSCT, with or without CAR T-cell therapy. On the *y*-axis, the odd ratio is represented with a baseline value of 1.00. The blue square and solid lines represent event rates with 95% confidence intervals. The size of the squares indicates the weight of each study. The black rhombus indicates the pooled estimate with 95% confidence intervals for each subgroup.

Similar to the MRD-CR-based efficacy, to determine the RRs after treatment, we divided the CAR T-cell therapy into three subgroups based on the target CD. In the RR analysis, a lower event rate was considered as a better clinical outcome. In this panel, we enrolled 17 studies for CD target analysis (Figure 3(A)). The results suggested that the anti-CD22 (2/17 studies) showed the lowest RR (ER = 0.24; 95% CI = 0.09, 0.40; *p* = 0.02; *I*^2^ = 53.72%); while anti-CD19 (12/17 studies) and the combination anti-CD19/CD22 (3/17 studies) showed RRs of 0.29 (95% CI = 0.24, 0.34; *p* < 0.01; *I*^2^ = 51.22%) and 0.58 (95% CI = 0.44, 0.72; *p* = 0.74; *I*^2^ = 77.06%), respectively (Figure 3(A)). Although we found higher RR in the anti-CD19/CD22 subgroup, the overall RR of this therapy in r/r B-ALL was 0.31 with a moderate heterogeneity among studies (95% CI = 0.27, 0.36*; p* < 0.01; I^2^ = 66.94) (Figure 3(A)).

Next, we analyzed the RRs involving the costimulatory domains of the anti-CD19 target group. We found that the CD28ζ (2/12 studies) CAR T-cell therapy showed the lowest RR (ER = 0.16; 95% CI = 0.04, 0.27; *p* < 0.01; *I*^2^ = 53.21%); followed by 4-1BB & CD3ζ (2/12 studies) (ER = 0.28; 95% CI = 0.20, 0.36; *p* < 0.01; *I*^2^ = 0.00%), CD3ζ & CD28ζ (1/12 studies) (ER = 0.33; 95% CI = 0.15, 0.51; *p* = 0.11), 4-1BB (6/12 studies) (ER = 0.35; 95% CI = 0.27, 0.44; *p* < 0.01; *I*^2^ = 0.00%), and the 4th generation CAR T-cell group (1/12 studies) (ER = 0.90; 95% CI = 0.57, 1.23). The pooled analysis of costimulatory domain targets showed a significant overall event rate and moderate heterogeneity (ER = 0.30; 95% CI = 0.25, 0.35; *p* < 0.01; *I*^2^ = 55.47%) (Figure 3(B)).

Additionally, we analyzed the effects of haploidentical haematopoietic stem cell transplantation (haplo-HSCT) post-CAR T-cell therapy on preventing further relapse of B-ALL, and the efficacy of haplo-HSCT without CAR T-cell therapy, which was reported in two included studies (Figure 3(C)). It was noted that CAR T-cell with haplo-HSCT might reduce the event of relapse significantly (OR = 3.38; 95% CI = 1.32, 8.69; *p* = 0.01), with the anti-CD19 (OR = 3.53; 95% CI 1.26, 9.88; *p* = 0.02) showing a better result compared to the anti-CD22 group (OR = 2.82; 95% CI = 0.28, 28.52; *p* = 0.38). Overall, the haplo-HSCT combined with CAR T-cell therapy showed more favourable results compared to haplo-HSCT alone in addressing the relapse issue in B-ALL patients, albeit non-significantly (OR = 1.78; 95% CI = 0.66, 4.74; *p* = 0.25).

#### Safety of CAR T-cell therapy for r/r B-ALL

##### CRS-based safety of CAR T-cell therapy

**Figure 4.**
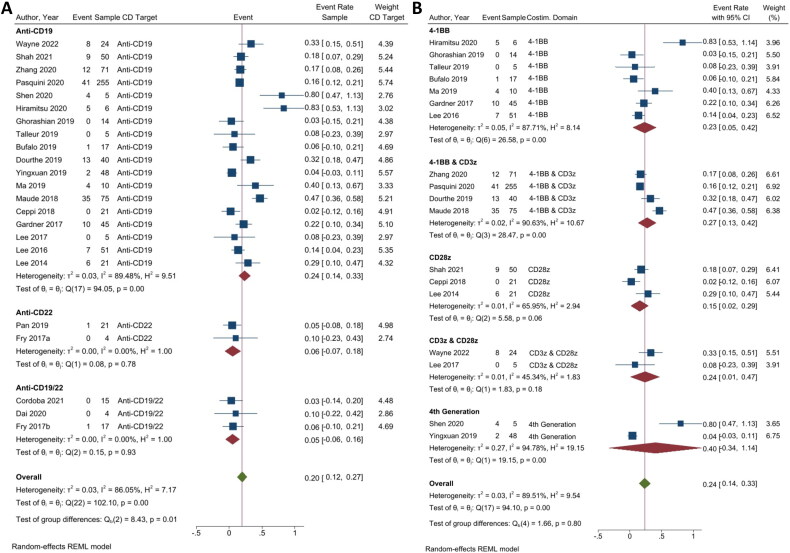
CAR T-cell safety. (A) Subgroup analysis of CD targets for CRS. The analysis included data from 23 studies classified into three CD target subgroups, namely anti-CD19, anti-CD22, and anti-CD19/22. (B) Subgroup analysis of costimulatory domain target groups for CRS. The analysis included data from 18 studies classified into five costimulatory subgroups, namely 4-1BB, 4-1BB & CD3ζ, CD28ζ, CD3ζ & CD28ζ and 4th generation. The *y*-axis indicates the median of the overall pooled estimate. The blue square and solid lines represent event rates with 95% confidence intervals. The size of the squares indicates the weight of each study. The red rhombus indicates the pooled estimate with 95% confidence intervals for each subgroup; meanwhile, the green rhombus indicates the overall pooled estimate.

CRS, a systemic inflammatory response caused by cytokines released by infused CAR T-cells, could lead to widespread reversible organ dysfunction. CRS is the most common type of toxicity caused by CAR T-cells. Thus, the lower event rate of CRS signified a more favourable outcome. Twenty-three studies were included in the CRS analysis to assess the safety of each group of CAR T-cell therapy. Two independent analyses were conducted, each focusing on the anti-CD19, CD22, or combination of CD19/CD22, and the anti-CD19 with costimulatory domain CAR T-cell therapy (Figure 4(A) and (B)).

CAR T-cell therapy utilizing the combination of anti-CD19/22 (3/23 studies) showed the lowest event rate of CRS (ER = 0.05; 95% CI = −0.06, 0.16; *p* < 0.01; *I*^2^ = 0.00%), followed by anti-CD22 (2/23 studies) (ER = 0.06; 95% CI = −0.07, 0.18; *p* < 0.01; *I*^2^ = 0.00%), and anti-CD19 (18/23 studies) (ER = 0.24; 95% CI = 0.14, 0.33; *p* < 0.01; *I*^2^ = 89.48%), respectively. The pooled quantitative analysis focusing on the occurrence of CRS in the anti-CD groups showed a significant overall event rate with a high heterogeneity (ER = 0.20; 95% CI = 0.12, 0.27; *p* < 0.01; *I*^2^ = 86.05%) (Figure 4(A)).

The analysis of anti-CD19 costimulatory domain showed that among six subgroups, the costimulatory domain with the least CRS event rate was CD28ζ (3/18 studies) (ER = 0.15; 95% CI = 0.02, 0.29; *p* < 0.01; *I*^2^ = 65.95%), followed by 4-1BB (7/18 studies) (ER = 0.23; 95% CI = 0.05, 0.42; *p* < 0.01; *I*^2^ = 87.71%), CD3ζ & CD28ζ (2/18 studies) (ER = 0.24; 95% CI = 0.01, 0.47; *p* = 0.12; *I*^2^ = 45.34%), 4-1BB & CD3ζ (4/18 studies) (ER = 0.27; 95% CI = 0.13, 0.42; *p* = 0.10; *I*^2^ = 90.63%), and 4th generation (2/18 studies) (ER = 0.40; 95% CI = −0.34, 1.41; *p* = 0.67; *I*^2^ = 94.78%). The pooled analysis of CRS in CD19 costimulatory domain CAR T-cell displayed a significant overall event rate with a high heterogeneity (ER = 0.24; 95% CI = 0.14, 0.33; *p* ≤ 0.01; *I*^2^ = 89.51%) (Figure 4(B)).

##### ICANS-based safety of CAR T-cell therapy

**Figure 5.**
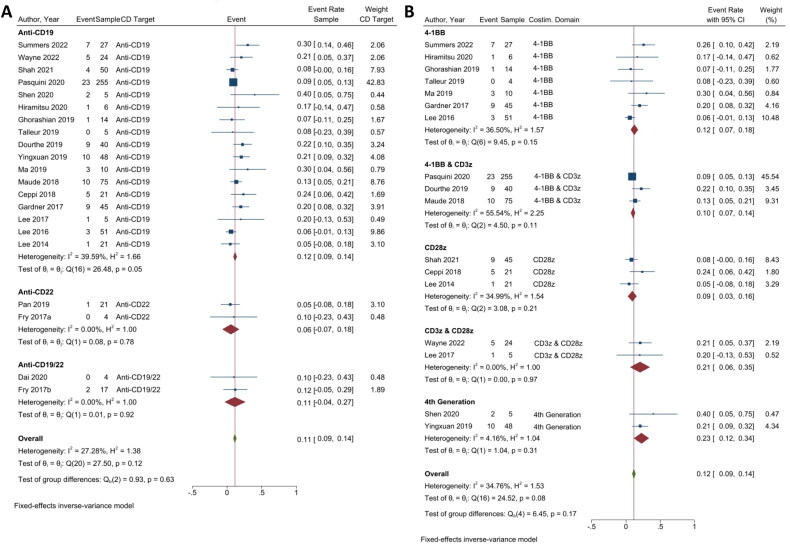
Immune effector cell-associated neurotoxicity syndrome in CAR T-cell therapy. (A) Subgroup analysis of CD targets for ICANS. The analysis included data from 21 studies classified into three CD target subgroups, namely anti-CD19, anti-CD22, and anti-CD19/22. (B) Subgroup analysis of costimulatory domain target groups for ICANS. The analysis included data from 17 studies classified into five costimulatory subgroups, namely 4-1BB, 4-1BB & CD3ζ, CD28ζ, CD3ζ & CD28ζ, and 4th generation. The *y*-axis indicates the median of the overall pooled estimate. The blue square and solid lines represent event rates with 95% confidence intervals. The size of the squares indicates the weight of each study. The red rhombus indicates the pooled estimate with 95% confidence intervals for each subgroup; meanwhile, the green rhombus indicates the overall pooled estimate.

A common and challenging side effect associated with CAR T-cell therapy is ICANS, which occurs in 20–60% of patients [46], of whom 12–30% showed severe (≥grade 3) symptoms. Similar to RR and CRS, a lower event rate in ICANS revealed a better treatment outcome. Two independent analyses were conducted, focusing on anti-CD CAR T-cell therapy (21 studies) and anti-CD19 costimulatory domains (17 studies) (Figure 5(A) and (B)).

Inversely from the CRS, anti-CD22 (2/21 studies) showed the lowest event rate of ICANS (ER = 0.06; 95% CI = −0.07, 0.18; *p* = 0.78; *I*^2^ = 0.00%), followed by anti-CD19/CD22 (2/21 studies) (ER = 0.11; 95% CI = −0.04, 0.27; *p* = 0.92; *I*^2^ = 0.00%), and anti-CD19 (17/21 studies) (ER = 0.12; 95% CI = 0.09, 0.14; *p* = 0.05; *I*^2^ = 39.59%). In this CD target group analysis, there was no significance in the overall event rate with a low overall heterogeneity (ER = 0.11; 95% CI = 0.09, 0.14; *p = 0.12*; *I*^2^ = 27.28%) (Figure 5(A)).

Similar to the CRS, among the five subgroups in the anti-CD19 costimulatory domain analysis, CD28ζ (3/17 studies) showed the lowest event rate of ICANS (ER = 0.09; 95% CI = 0.03, 0.16; *p* = 0.21; *I*^2^ = 34.99%), followed by 4-1BB & CD3ζ (3/17 studies) (ER = 0.10; 95% CI = 0.07, 0.14; *p =* 0.11; *I*^2^ = 55.54%), 4-1BB (7/17 studies) (ER = 0.12; 95% CI = 0.07, 0.18; *p =* 0.15; *I*^2^ = 36.50%), CD3ζ & CD28ζ group (2/17 studies) (ER = 0.21; 95% CI = 0.06, 0.35; *p* = 0.97; *I*^2^ = 0.00%), and 4th generation (2/17 studies) (ER = 0.23; 95% CI = 0.12, 0.34; *p* = 0.31; *I*^2^ = 4.16%), respectively. In this analysis, a significant overall event rate was also observed, with a statistical homogeneity (ER = 0.12; 95% CI = 0.09, 0.14; *p* = 0.17; *I*^2^ = 34.76%) (Figure 5(B)).

## Discussion

To date, the treatment of paediatric patients with r/r B-ALL remains a global challenge as reflected by the significant events of relapses and reduction of long-term survival to 30–60% [3]. Therapeutic advancements have sprung in the field of immunotherapy in the last decades, which were specifically designed to target specific cells to enhance the therapeutic efficacy and recovery. The concept of CAR T-cell therapy was first introduced in 1989 and had since undergone swift developments. After over three decades of research, CAR T-cell therapy has demonstrated more favourable outcomes in treating haematologic tumours compared to other existing immunotherapies [47]. Moreover, the combination of CAR T-cell therapy with haplo-HSCT has also displayed enhanced effectiveness in several studies [17, 37, 45]. Nevertheless, this treatment remains controversial due to relapses in certain individuals and the occurrence of immune-related severe adverse events, such as high-grade CRS and ICANS. Selecting a safer and more effective CAR T-cell target and costimulatory domains could potentially improve the overall survival and enhance the safety in r/r B-ALL patients.

In this meta-analysis, we found that the efficacy of the newly developed anti-CD22 CAR T-cell was comparable to that targeting CD19. Meanwhile, the supposedly promising anti-CD19/22 was less efficacious despite showing a comparable safety profile to anti-CD22 CAR T-cell, both of which were safer than the commonly used anti-CD19. Furthermore, the analysis of CD19 costimulatory receptors showed that the addition of CD28ζ to anti-CD19 CAR T-cell was superior in reducing the RR with the best safety profiles.

The efficacy of CAR T-cell therapy for r/r B-ALL using different approaches revealed that targeting both the CD22 and CD19 showed a positive trend of remission, as reflected by MRD-CR. Despite the smaller number of studies included, the anti-CD22 showed an equivalent event rate of MRD-CR as the widely used anti-CD19. To address this, a higher percentage of patients (51/55 patients (92.7%)), who had previously experienced failure with CD19 CAR T-cells, were now receiving CD22 CAR-T therapy as a second-line treatment. In another study, patients were considered eligible if they had relapsed or refractory CD22-positive B-ALL and no curative treatment options, including CD19 CAR-T therapy [37]. As a result, the effectiveness of anti-CD22 therapy may be evaluated as more potent than the prior anti-CD19 treatment.

Besides the main CD target, the costimulatory domains are required to achieve a robust CAR-T function. This study revealed that the combination of 4-1BB and CD3ζ to anti-CD19 CAR T-cells showed the highest MRD-CR. A prior meta-analysis analyzing the efficacy of CD19 costimulatory domains showed that anti-CD19 CAR T-cell therapy with 4-1BB domain was the most efficacious, followed by the 4th generation and CD28ζ domains [48]. The slight variations of the findings may arise from the differences of analyzed costimulatory domains in each of the studies. Other than that, our findings also stated the performance of 4-1BB in combination with CD3ζ was superior to CD28ζ. On the other hand, CD28ζ showed a higher MRD-CR rate compared to 4-1BB alone. Our finding was in accordance with a review from Cappell et al. where CD28ζ was superior to 4-1BB in their CAR T-cells activity [49]. However, it has also been noted that two studies included in the review reported the superiority of 4-1BB, which was in accordance with the findings of Myers et al. [50]. In our study, the increased efficacy of 4-1BB is likely due to the nature of CD3ζ, where it has been reported that the addition of CD3ζ in 4-1BB signalling domain resulted in higher antigen-induced clonal expansion, as well as higher tumouricidal activity *in vitro* as stated by Zhong et al. [51]. These authors also noted the superiority of CD28ζ as a costimulatory domain that increased the tumouricidal activity. Nevertheless, the precedence of either CD28ζ or 4-1BB in clinical settings remains inconsistent depending on the immune response of CAR T-cell in individual patients. It is also interesting to note the possibility of CD28ζ’s combination with other costimulatory domains having better responses due to its similar activity with CD3ζ as described in the same study [51].

The bispecific anti-CD19/CD22 CAR T-cell was developed to tackle the antigenic escape using the dual targeting strategy. However, our analysis revealed that this approach was inferior compared to the anti-CD19 or anti-CD22 groups in achieving MRD-CR. Conversely, another meta-analysis discovered a notably higher CR rate when using anti-CD19/CD22 CAR T-cell therapy in comparison to the use of single-target CD22 CAR T-cells [52]. Nevertheless, it is still early to state the conclusion regarding the performance of anti-CD19/CD22 due to the limited number of studies.

Reduction in RR is one of the main targets of CAR T-cell therapy. Regardless of the number of studies and variation of patient profiles, we found that the RR yielded significantly favourable outcomes towards anti-CD22 CAR T-cells therapy compared to those of anti-CD19. This finding was supported by a study by Fry and colleagues, which reported that the administration of anti-CD22 CAR T-cells resulted in a lower incidence of relapse compared to anti-CD19 [38]. This was possibly caused by the rise of CD19 negative relapse issue in r/r B-ALL patients, which emerged as the dominant mechanism of resistance towards this class of therapy. However, the majority of them retained the CD22 expression, making it a potent target.

It is noteworthy that, in addition to their inferior performance in MRD-CR, the bispecific anti-CD19/CD22 also failed to display promising outcomes in RRs when compared to the individual anti-CD groups. In a study conducted by Wang et al., the administration of the anti-CD19/CD22 CAR T-cells resulted in only a brief longevity of both CAR T-cell populations [53]. Another study by Maude et al. noted the high RR in anti-CD19/22 CAR T-cell therapies [54].

The reduced efficacy of the bispecific anti-CD19/22 CAR T-cell therapy could stem from the differences in the vector design, specifically the selection of the promoter. A study by Shalabi et al. revealed that individuals receiving anti-CD22 CAR T-cells with the elongation factor 1α (EF1α) promoter experienced significantly higher peak expansion in peripheral blood and bone marrow compared to those who received anti-CD19/22 CAR T-cells with the murine stem cell virus (MSCV) promoter, although further laboratory investigations comparing these promoters did not show a significant disparity in CAR T-cell efficacy. Furthermore, the study found that anti-CD19/22 CAR T-cell therapy resulted in a high percentage of CAR-expressing CD8+ T-cells displaying an exhaustion phenotype by day 42, as indicated by the expression of PD1 and Tim3 [55]. At the basal state, nonactivated T-cells transmits a low-level constitutive signal known as a tonic TCR signal in the absence of ligands, contributing to cell differentiation and maintenance of cellular responses to antigen stimulation. In CAR T-cell therapy, the self-aggregation of the receptor triggers different degrees of ligand-independent signalling referred to as tonic signal, and increased levels of this signal have been associated with the exhaustion and impaired function of CAR T-cells [56].

Among the CD19 costimulatory domain analyzed, CD28ζ, resulted in the lowest RR. This was consistent with previous evidence that the CAR costimulatory domain, CD28ζ, played a dominant role in modulating the rate of expansion and the likelihood of persistence following CAR T-cell therapy [57]. An *in vitro* functional assays showed that CD28ζ CAR T-cells induced higher levels of released cytokines such as IL-2, IFN-γ, and TNFα, and an enhanced cytotoxic effect than 4-1BBζ and CD3ζ CAR T-cell [58]. Kinetics and protein phosphorylation profile studies also showed that CD28ζ CAR was more prone to activate effector T-cell-associated genes, while 4-1BBζ and CD3ζ CARs preferentially activated memory T-cell-associated genes [59]. Although the addition of CD28ζ costimulatory seemed to be superior in efficacy, this might raise concerns of the safety profiles due to the high cytokine production and release. Overall, this analysis might highlight the importance of 4-1BB, 4-1BB & CD3ζ, CD28ζ, and 4th generation costimulatory domains in anti-CD19 CAR T-cell therapy as further intervention of interests in developing more efficacious CAR T-cell therapy.

Inversely to the efficacy evaluations, the safety analysis of CRS showed that the combination of anti-CD19/22 CAR T-cell therapy resulted in the least CRS event, which aligned with a previous meta-analysis [52]. A retrospective study comparing the severe CRS of the single anti-CD19 and the combination of anti-CD19/22 discovered that significantly more patients developed severe CRS in the anti-CD19 group [60]. Aligned with the previous theory regarding tonic TCR signalling, increased tonic TCR signals observed in anti-CD19/22 CAR T-cell therapy led to upregulation of inhibitory receptor genes, such as lymphocyte-activation gene (*LAG*) and cytotoxic T-lymphocyte-associated protein 4 (*CTLA4)*, and downregulation of memory-related genes (lymphoid enhancer-binding factor 1 [*LEF1*], T-cell factor 7 [*TCF7*], interleukin 7 receptor [*IL7R*], and Kruppel-like factor 2 [*KLF2*]), which might explain the lesser occurrence of severe CRS in this CAR T-cell type [56].

Our analysis showed that haplo-HSCT in combination with CAR T-cell therapy was favourable in reducing the RR of B-ALL when compared to HSCT alone, although the number of studies addressing this issue was limited. Moreover, when combined with haplo-HSCT, the administration of anti-CD19 CAR T-cells resulted in a lower incidence of relapse compared to anti-CD22. This might be explained since CD19 is considered as a pan-B-cell marker, as it is expressed on B-cells from the earliest recognizable B-lineage cells during development [61]. CD19 expression at the earliest precursor stage of B-cells allowed more cells to interact with anti-CD19 CAR T-cells. HSCT makes the functional CAR T-cells present for a relatively longer period, enabling more functional CAR T-cells to persist with HSCT compared to CD22 [62]. The duration of CAR T-cell persistence has been indicated to be critical for preventing relapse incidence [63]. Future efforts to understand the mechanisms affecting the duration of CAR T-cell persistence are warranted to further improve the CAR T-cell persistence and ensuring lower RRs after CAR T-cell infusion.

In addition, CAR T-cell therapy after haplo-HSCT transplant might enable more patients to achieve MRD-CR by providing them the opportunity to bridge into a second transplant with HSCT. B-ALL patients who relapsed after the first transplant and achieved remission with CAR T-cell therapy, followed by consolidation of secondary haplo-HSCT, have been reported to be associated with longer survival [29]. Unfortunately, transplant-associated thrombotic microangiopathy (TA-TMA) was one of the top three causes of death in this combination regiment and secondary haplo-HSCT poses as a risk for the incidence of TA-TMA [64].

Aside from haplo-HSCT transplantation, allogeneic haematopoietic stem cell transplantation (allo-HSCT) has emerged as a viable option for patients showing resistance to induction chemotherapy or experience early relapse following initial therapy [65]. A recent investigation conducted by Cao et al. aimed to assess the efficacy of employing a second allo-HSCT following CAR T-cell therapy in patients with r/r B-ALL [46]. Their findings revealed that integrating CAR-T therapy with a subsequent consolidation allo-HSCT significantly enhanced the observed outcomes, with MRD-CR of 90.5% and RR of 10.5% in individuals with r/r B-ALL and post-transplant relapse. These outcomes were relatively superior to those without the combination of a second allo-HSCT and CAR T-cell therapy. However, there is limited information concerning the specific analysis of allo-HSCT following CAR T-cell therapy in paediatric patients with r/r B-ALL. This underscores the need for future research to delve into the potential benefits and outcomes of allo-HSCT after CAR T-cell therapy in paediatric r/r B-ALL populations.

Future research should explore a broader range of CD targets for CAR T-cell therapy. Various novel CD targets, such as CD19.28ζ/CD22.BBζ and CD99, have demonstrated high efficacy in preclinical models. The CD19.28ζ/CD22.BBζ CAR promoted by EF1α exhibited an enhanced capacity for CD22 targeting, thereby promoting the persistence and expansion of T-cells [55, 66, 67]. Recent advancements in immunotherapy utilizing neoantigen-reactive T-cells (NRT cells) also have achieved notable success in treating melanoma and various solid tumours. Neoantigens, resulting from non-synonymous mutations in tumour genes, can trigger the activation of neoantigen-reactive T-cells (NRT), demonstrating the capability to impede the growth of tumours expressing specific neoantigens [68]. Furthermore, investigations are warranted to assess the potential of employing NRT cells in the treatment of haematologic malignancies. Over the years, T-cell-based cancer-targeting immunotherapies targeting antigens has been the focus of research and development. Identifying multiple targeted antigens and developing T-cell-based therapies that express multiple antigen-specific CARs or TCRs could overcome the tumour heterogeneity and increase effectiveness. Furthermore, parts of a tumour may escape the immune response by upregulating check-point molecules that suppresses the effector T-cell function or by losing HLA expression, causing a deficiency in the antigen processing and presentation machinery. Combination therapies targeting tumour antigens along with check-point inhibitors or the use of alternative cell sources, such as NK cells, MAIT cells, and γδ T-cells, may offer solutions [69].

To the best of our knowledge, this article serves as the latest systematic review on this area since Aamir et al. in 2020 and the CMA by Fergusson et al. in 2023, which described the efficacy and safety of CAR T-cells targeting CD22 and CD19/22 [48, 52]. Here, we provided a thorough analysis of the efficacy and safety of CAR T-cell therapy in r/r B-ALL by displaying the subgroup analyses from individual anti-CD19, anti-CD22, and the bispecific antiCD19/CD22 targets. Additionally, we provided the information on the anti-CD19 costimulatory domain targets in each study parameter. With the fast growing in the field of CAR T-cell therapy, other systematic reviews and meta-analysis in the future might yield different results due to the addition of studies focusing on other anti-CD subgroups and more costimulatory domains. In the future, their combination therapy of CAR T-cell and haplo-HSCT might be worth to be discussed further.

## Conclusion

The management of r/r B-ALL remains a great challenge. The widely utilized chemotherapy is less efficacious in preventing relapse in r/r B-ALL, resulting in high mortality rates among paediatric populations. CAR T-cell therapy as a novel approach to r/r B-ALL proved to be promising in managing this condition. The currently widely used CAR T-cell therapy targeting CD19, or those involving certain CD19 costimulatory domains, still showed acceptable performance in efficacy and safety, although the newly developed anti-CD22 CAR T-cell provided the best efficacy. The combination of CAR T-cell and haplo-HSCT also displayed favourable results, suggesting its potential for wider use in future clinical applications. Furthermore, it is imperative for future studies to investigate the potential advantages and outcomes of employing allo-HSCT following CAR T-cell therapy in paediatric patients with r/r B-ALL, as well as to explore a wider array of CD targets for CAR T-cell therapy.

## Supplementary Material

Supplemental Material

## Data Availability

Data available within the article or its Supplementary Materials.
