# Supplementary material for: Comprehensive analysis of the efficacy and safety of CAR T-cell therapy in patients with relapsed or refractory B-cell acute lymphoblastic leukaemia: a systematic review and meta-analysis
Source: Ann Med. 2024 May 13;56(1):2349796. doi: 10.1080/07853890.2024.2349796 (PMC11095278; doi:10.1080/07853890.2024.2349796)
Supplement: Supplemental Material [file IANN_A_2349796_SM9972.docx]

**Supplementary Table 1.** PICO framework

| **Patient** | Pediatric patients with relapse or refractory B-cell acute lymphoblastic leukemia (r/r B-ALL) |
| --- | --- |
| **Intervention** | anti-CD19, anti-CD22, and combination of anti-CD19/22 CAR T-cell therapy |
| **Control** | Haploidentical hematopoietic stem cell transplant (haplo-HSCT) and chemotherapy |
| **Outcome** | Minimal complete residual disease negative complete remission (MRD- CR), relapse rate (RR), cytokine release syndrome (CRS), and immune effector cell-associated neurotoxicity syndrome (ICANS) |

**Supplementary Table 2.** Newcastle-Ottawa Scale (NOS) analysis for risk of bias assessment

| **No** | **Author, Year** | **Selection Domain** |  |  |  | **Comparability** | **Outcome Domain** |  |  | **Total score** |
| --- | --- | --- | --- | --- | --- | --- | --- | --- | --- | --- |
|  |  | **Representativeness of the Exposed** | **Selection of the Non-Exposed** | **Ascertainment of Exposure** | **Outcome Negative at Start** |  | **Assessment of Outcome** | **Enough Follow-Up Period** | **Adequacy of Follow Up** |  |
| 1 | Jiang *et al.*, 2021 | * | * | * | * | * | * | * | * | 8 |
| 2 | Summers *et al.*, 2022 | * | * | * | * | * | * | * | * | 8 |
| 3 | Hu *et al.*, 2022 | * | * | * | * | * | * | * | * | 8 |
| 4 | Zhao *et al.*, 2021 | * | * | * | NR | NR | * | * | * | 6 |
| 5 | Pan *et al.*, 2019 | * | * | * | * | ** | * | * | * | 9 |
| 6 | Fry *et al.*, 2018 | * | * | * | * | NR | * | * | * | 7 |
| 7 | Curran *et al.*, 2019 | * | * | * | NR | ** | * | * | * | 8 |
| 8 | Maude *et al.*, 2018 | * | NR | * | * | * | * | * | * | 7 |
| 9 | Levin *et al.*, 2021 | * | NR | * | * | ** | * | * | * | 8 |
| 10 | Shah *et al.*, 2021 | * | * | * | NR | NR | * | * | * | 6 |
| 11 | Gardner *et al.*, 2017 | * | * | * | NR | * | * | * | * | 7 |
| 12 | Ghora *et al.*, 2019 | NR | NR | * | NR | NR | * | * | * | 4 |
| 13 | Lee *et al.*, 2014 | * | * | * | * | ** | * | * | * | 9 |
| 14 | Ma *et al.*, 2019 | * | * | * | * | * | * | * | * | 8 |
| 15 | Wayne *et al.*, 2022 | * | * | * | NR | NR | * | * | * | 6 |
| 16 | Leahy *et al.*, 2019 | * | NR | * | * | * | NR | * | * | 6 |
| 17 | Lee *et al.*, 2017 | * | NR | * | NR | NR | NR | * | * | 4 |
| 18 | Talleur *et al.*, 2019 | NR | NR | * | NR | * | NR | * | * | 4 |
| 19 | Hiram *et al.*, 2020 | * | NR | * | * | * | * | * | * | 7 |
| 20 | Lee *et al.*, 2016 | * | * | * | NR | * | * | * | * | 7 |
| 21 | Zhang *et al.*, 2020 | * | * | * | NR | * | * | * | * | 7 |
| 22 | Ceppi *et al.*, 2018 | * | * | * | NR | * | * | * | * | 7 |
| 23 | Bufalo *et al.*, 2019 | * | * | * | * | * | * | * | * | 8 |
| 24 | Dourthe *et al.*, 2019 | * | * | * | * | * | * | * | * | 8 |
| 25 | Yingxuan *et al.*, 2019 | * | NR | * | * | * | NR | * | * | 6 |
| 26 | Shen *et al.*, 2020 | NR | * | * | * | ** | * | * | * | 8 |
| 27 | Dai *et al.*, 2020 | NR | * | * | * | ** | * | * | * | 8 |
| 28 | Cordoba *et al.*, 2021 | * | NR | * | * | ** | * | * | * | 8 |
| 29 | Pasquini *et al.*, 2020 | * | * | * | * | * | * | * | * | 8 |

Star indicates the scores of each domain; for the domain of comparability, (*) if only one item of either same age or other controlled factors are fulfilled, or (**) if both items are fulfilled, NR: not reported

**Supplementary Table 3**. Characteristics of included studies

| **No** | **Author, Year** | **Study Design**  **(Center)** | **Duration**  **(Months)** | **Participant characteristics** |  | **CAR T-cell intervention** |  | **Efficacy** |  | **Safety** |  |
| --- | --- | --- | --- | --- | --- | --- | --- | --- | --- | --- | --- |
|  |  |  |  | **Subject**  **(M: F)** | **Median age (Year)**  **(range)** | **Type** | **Dose (cells/kg)**  **(range)** | **RR** | **MRD- CR** | **CRS** | **ICANS** |
| 1 | Jiang *et al*., 2021 | Treatment Trials (single) | 1 | 37  (21:16) | NR | Anti-CD19  (CD137 – 2^nd^ gen) | 3.93 x 10^6^  (0.47–8.63 x 10^6^) | NR | 78.38%  (29/37) | NR | NR |
| 2 | Summers *et al*., 2022 | Cohort  (multiple) | 48 | 27  (17:10) | 15 (1-25) | Anti-CD19  (4-1BB)^a^ | 1 x 10^6^ | 29.63% (8/27) | 48.15%  (13/27) | NR | 25.93%  (7/27) |
|  |  |  |  | 23  (10:13) | 12 (1-22) | Anti-CD19  (4-1BB)^b^  (Fractionated total body irradiation (TBI), busulfan, melphalan) | * | 21.74%  (5/23) | 56.52%  (13/23) | NR | 21.74% (5/23) |
| 3 | Hu *et al.*, 2022 | Cohort  (single) | 36 | 14  (9:5) | 9 (3-18) | Haplo-HSCT | NR | 50.00%  (7/14) | 35.71%  5/14 | NR | NR |
|  |  |  |  | 26  (16:12) | 9.5 (1-18) | Anti-CD19  (4-1BB)^b^ | 4.04 x 10^6^  (0.35–6.51 x 10^6^) | 26.92%  (7/26) | 88.46%  (23/26) | 57.69%  (15/26) | 11.54%  (3/26) |
| 4 | Zhao *et al*., 2021 | Cohort  (single) | 36 | 78  (11:67) | 14.5 (2-30) | Haplo-HSCT | NR | 14.93%  (10/67) | NR | 25.37%  (17/67) | NR |
|  |  |  |  | 27  (4:23) | 11 (3-30) | Anti-CD19  (4-1BB)^b^ | NR | 13.04%  (3/23) | NR | 47,83%  (11/23) | NR |
| 5 | Pan *et al*., 2019 | MAMS Trials  (single) | 12 | 21  (14:7) | 9 (1-25) | Anti-CD22  (4-1BB & CD3ζ) | >4 x 10^6^ | 4/21 (19.05%) | 80.95%  (17/21) | 4.76%  (1/21) | 4.76%  (1/21) |
|  |  |  |  | 13  (6:7) | 11 (2-29) | Anti-CD22  (4-1BB & CD3ζ)^b^ | >4 x 10^6^ | 1/13 (7.69%) | 92.31%  (1/21) | 0%  (0/13) | 0%  (0/13) |
| 6 | Fry *et al*., 2017 | MAMS Trials  (single) | 24 | 4  (2:2) | 16.5 (12-19) | anti-CD22  (4-1BB & CD3ζ) | 1 x 10^6^ | 50.00%  (2/4) | 50.00%  (2/4) | 0%  (0/4) | 0%  (0/4) |
|  |  |  |  | 17  (11:6) | 18.18 (7-30) | Anti-CD19  (4-1BB) & Anti-CD22  (4-1BB & CD3ζ) | 1 x 10^6^ | 35.29%  (6/17) | 41.18%  (7/17) | 5.88%  (1/17) | 11.76%  (2/17) |
| 7 | Curran *et al*., 2019 | Treatment Trials (multiple) | 24 | 25  (NR) | 13.5  (1-22.5) | Anti-CD19  (CD28ζ)^b^ | NR | 16.00%  (4/25) | 64.00%  (16/25) | 16.00%  (4/25) | 28.00%  (7/25) |
|  |  |  |  |  |  |  |  |  |  |  |  |
| 8 | Maude *et al*., 2018 | Cohort  (multiple) | 24 | 75  (NR) | 11  (3-23) | Anti-CD19  (4-1BB and CD3ζ) | 3.1 x 10^6^  (0.2–5.4 x10^6^) | 29.33%  (22/75) | 77.33%  (58/75) | 46.67%  (35/75) | 13.33%  (10/75) |
|  |  |  |  |  |  |  |  |  |  |  |  |
| 9 | Levin *et al*., 2021 | Treatment Trials  (multiple) | 24 | 137  (72:65) | 12  (3-25) | Anti-CD19  (4-1BB &CD3ζ) | 1 x 10^8^  (0.03–2.6 x 10^8^) | NR | NR | 41.61%  (57/137) | 10.22%  (14/137) |
|  |  |  |  |  |  |  |  |  |  |  |  |
| 10 | Shah *et al*., 2021 | Treatment Trials  (single) | 24 | 50  (40:10) | 13.5  (4.3-30.4) | Anti-CD19  (CD28ζ) | 1 x 10^6^ | NR | 56.00%  (28/50) | 18.00%  (9/50) | 8.00%  (4/50) |
|  |  |  |  |  |  |  |  |  |  |  |  |
| 11 | Gardner *et al*., 2017 | Treatment Trials  (multiple) | 21 | 45  (23:22) | 12.2  (1.3-25.3) | Anti-CD19  (4-1BB) | 0.5-10 x 10^6^ | 40.00%  (18/45) | 88.89%  (40/45) | 22.22%  (10/45) | 20.00%  (9/45) |
|  |  |  |  |  |  |  |  |  |  |  |  |
| 12 | Ghorashian *et al*., 2019 | Treatment Trials  (single) | 24 | 14  (NR) | 9 (1-24) | Anti-CD19  (4-1BB) | 1.2 x 10^6^ | 42.86%  (6/14) | 85.71%  (12/14) | 0.00%  (0/14) | 7.14%  (1/14) |
|  |  |  |  |  |  |  |  |  |  |  |  |
| 13 | Lee *et al*., 2014 | Treatment Trials  (single) | 24 | 21  (14:7) | 11.5 (5-27) | Anti-CD19  (CD28ζ) | 1 x 10^6^ | 9.52%  (2/21) | 57.14%  (12/21) | 28.57%  (6/21) | 4.76%  (1/21) |
|  |  |  |  |  |  |  |  |  |  |  |  |
| 14 | Ma *et al*., 2019 | Treatment Trials  (single) | 18 | 10  (4:6) | 6.5 (3-13) | Anti-CD19  (4-1BB)^a^ | 0.7 x 10^6^  (0.3‐1.58 x 10^6^) | 40.00%  (4/10) | 40.00%  (4/10) | 40.00%  (4/10) | 30.00%  (3/10) |
| 15 | Wayne *et al*., 2022 | Treatment Trials  (multiple) | 43 | 24  (15:9) | 13.5 (3-20) | Anti-CD19  (CD3ζ & CD28)^a^ | 1-2 x 10^6^ | 33.00%  (8/24) | 38.00%  (9/24) | 33.00%  (8/24) | 21.00%  (5/24) |
| 16 | Leahy *et al*., 2019 | Treatment Trials  (single) | 31 | 111  (65:46) | 11 (1-29) | Anti-CD19  (4-1BB)^a^ | NR | NR | NR | NR | NR |
| 17 | Lee *et al*. 2017 | Treatment Trials  (single) | NR | 5  (NR) | 13.5 (2-21) | Anti-CD19  (CD3ζ & CD28)^a^ | 1.2 x 10^6^ | NR | 80.00%  (4/5) | 0.00%  (0/5) | 20.00%  (1/5) |
| 18 | Talleur *et al*., 2019 | Treatment Trials  (single) | NR | 4  (NR) | 11 (3-21) | Anti-CD19  (4-1BB)^a^ | 1 x 10^6^ | NR | 75.00%  (3/4) | 0.00%  (0/5) | 0.00%  (0/5) |
| 19 | Hiramatsu *et al*., 2020 | Treatment Trials  (multiple) | 60 | 6  (4:2) | 12.5 (5-24) | Anti-CD19  (4-1BB)^a^ | 0.2-5 x 10^6^ | 33.00%  (2/6) | 17.00%  (1/6) | 83.00%  (5/6) | 17.00%  (1/6) |
| 20 | Lee *et al*., 2016 | MAMS Trials | Median follow up 18.7 | 51  (NR) | NR | Anti-CD19  (4-1BB)^a^ | 1 x 10^6^ | NR | 55.00%  (28/51) | 13.70%  (7/51) | 5.80%  (3/51) |
|  |  |  |  | 21  (NR) | NR | Anti-CD19  (4-1BB) ^a^ | 1 x 10^6^ | 9.50%  2/21) | NR | NR | NR |
|  |  |  |  | 7  (NR) | NR | Anti-CD19  (4-1BB) ^b^ | 1 x 10^6^ | 85.70%  (6/7) | NR | NR | NR |
| 21 | Zhang *et al*., 2020 | Treatment Trials  (single) | NR | 71  (NR) | 12 (2-14) | Anti-CD19  (4-1BB + CD-28)^a^ | 3 x 10^6^  (0.2-10 x 10^6^) | NR | NR | 16.90%  (12/71) | 12.70%  (9/71) |
| 22 | Ceppi *et al*., 2018 | Treatment Trials  (NR) | 18 | 21  (NR) | 13  (10 - 17) | Anti-CD19  (CD28ζ) | 1 x 10^6^ | 27.70%  (5/18) | 86.00%  (18/21) | 0.00%  (0/21) | 24.00%  (5/21) |
| 23 | Bufalo *et al*., 2019 | Treatment Trials  (single) | 18 | 15  (NR) | 10  (1 - 25) | iC9-anti-CD19  (4-1BB - 2^nd^ gen.) | 3 x 10^6^ | 26.60%  (4/15) | 86.7.0%  (13/15) | 5.00%  (1/17) | NR |
| 24 | Dourthe *et al*., 2019 | Cohort  (multiple) | 18 | 40  (NR) | 18.2  (1 - 29.2) | Anti-CD19  (4-1BB & CD3ζ – 2^nd^ gen,) | 2.5 x 10^6^ | 26.30%  (10/38) | 87.50%  (35/40) | 32.50%  (13/40) | 22.50%  (9/40) |
| 25 | Yingxuan, et al., 2019 | Cohort  (NR) | 8 | 48  (29:19) | 8  (1 - 17) | Anti-CD19  (4^th^ gen.) | NR | NR | 35/48 (72.9%) | 2/48 (4%) | 10/48 (20%) |
| 26 | Shen *et al*., 2020 | Cohort  (single) | 5 | 5  (2:3) | 2  (1 - 12) | Anti-CD19  (4^th^ gen.) | NR | 100.00%  (4/4) | 80.00%  (4/5) | 80.00%  (4/5) | 40.00%  (2/5) |
| 27 | Dai *et al*., 2020 | Treatment Trials  (single) | 10 | 4  (3:1) | 21.5  (17 - 24) | Anti-CD19/CD22 | 3 x 10^6^ | 100.00%  (4/4) | 100.00%  (4/4) | 0.00%  (0/4) | 0.00%  (0/4) |
| 28 | Cordoba *et al*., 2021 | Treatment Trials  (multiple) | 12 | 15  (11:4) | 8  (4 – 16) | Anti-CD19/CD22 | 0.2-5 x 10^6^ | 69.20%  (9/13) | NR | 0.00%  (0/15) | (33.00%  (5/15) |
| 29 | Pasquini *et al*., 2020 | Cohort Studies  (multiple) | 12 | 255  (150:105) | 13.2  (1 - 26) | Anti-CD19  (4-1BB & CD3ζ - 2^nd^ gen.) | 0.2-5 x 10^6^ | NR | 99.1%  (115/116) | 16.1%  (41/255) | 9.00%  (23/255) |

CAR: chimeric antigen receptor, CRS: cytokine release syndrome, F: female, Gen.: generation, Haplo-HSCT: haploidentical hematopoietic stem cell transplantation, ICANS: Immune Effector Cell-Associated Neurotoxicity Syndrome, M: male, MAMS trials: multi-arm multi-stage trial, MRD- CR: minimal residual disease negative complete remission, NR: not reported, RR: relapse rate

^a^ CAR-T cell without Haplo-HSCT, ^b^ CAR-T cell with Haplo-HSCT, Star indicates the dose of the HSCT used (> 8 Gy, > 9.0 mg/kg, ≥ 150 mg/m^2^)
